# Correction: Povo-Retana et al. Trabectedin and Lurbinectedin Modulate the Interplay between Cells in the Tumour Microenvironment—Progresses in Their Use in Combined Cancer Therapy. *Molecules* 2024, *29*, 331

**DOI:** 10.3390/molecules30112472

**Published:** 2025-06-05

**Authors:** Adrián Povo-Retana, Rodrigo Landauro-Vera, Carlota Alvarez-Lucena, Marta Cascante, Lisardo Boscá

**Affiliations:** 1 Instituto de Investigaciones Biomédicas Alberto Sols-Morreale (CSIC-UAM), Arturo Duperier 4, 28029 Madrid, Spain; rlandauro@iib.uam.es (R.L.-V.); clucena@iib.uam.es (C.A.-L.); 2 Department of Biochemistry and Molecular Biomedicine-Institute of Biomedicine (IBUB), Faculty of Biology, Universitat de Barcelona, 08028 Barcelona, Spain; martacascante@ub.edu; 3 Centro de Investigación Biomédica en Red de Enfermedades Hepáticas y Digestivas (CIBEREHD), Instituto de Salud Carlos III (ISCIII), 28029 Madrid, Spain; 4 Centro de Investigación Biomédica en Red de Enfermedades Cardiovasculares (CIBERCV), Institute of Health Carlos III (ISCIII), 28029 Madrid, Spain

## Error in Affiliation

In the published publication [1], there was an error regarding the affiliation(s) for Marta Cascante. Instead of the published affiliation 3 text (Department of Material Science and Physical Chemistry, Research Institute of Theoretical and Computational Chemistry (IQTCUB), University of Barcelona, 08028 Barcelona, Spain), the updated affiliation 3 text should be the following: Centro de Investigación Biomédica en Red de Enfermedades Hepáticas y Digestivas (CIBEREHD), Instituto de Salud Carlos III (ISCIII), 28029 Madrid, Spain. 

The authors state that the scientific conclusions are unaffected. This correction was approved by the Academic Editor. The original publication has also been updated.
